# What Determines Auditory Distraction? On the Roles of Local Auditory Changes and Expectation Violations

**DOI:** 10.1371/journal.pone.0084166

**Published:** 2014-01-06

**Authors:** Jan P. Röer, Raoul Bell, Axel Buchner

**Affiliations:** Heinrich Heine University Düsseldorf, Düsseldorf, Germany; University of Salamanca- Institute for Neuroscience of Castille and Leon and Medical School, Spain

## Abstract

Both the acoustic variability of a distractor sequence and the degree to which it violates expectations are important determinants of auditory distraction. In four experiments we examined the relative contribution of local auditory changes on the one hand and expectation violations on the other hand in the disruption of serial recall by irrelevant sound. We present evidence for a greater disruption by auditory sequences ending in unexpected steady state distractor repetitions compared to auditory sequences with expected changing state endings even though the former contained fewer local changes. This effect was demonstrated with piano melodies (Experiment 1) and speech distractors (Experiment 2). Furthermore, it was replicated when the expectation violation occurred after the encoding of the target items (Experiment 3), indicating that the items' maintenance in short-term memory was disrupted by attentional capture and not their encoding. This seems to be primarily due to the violation of a model of the specific auditory distractor sequences because the effect vanishes and even reverses when the experiment provides no opportunity to build up a specific neural model about the distractor sequence (Experiment 4). Nevertheless, the violation of abstract long-term knowledge about auditory regularities seems to cause a small and transient capture effect: Disruption decreased markedly over the course of the experiments indicating that participants habituated to the unexpected distractor repetitions across trials. The overall pattern of results adds to the growing literature that the degree to which auditory distractors violate situation-specific expectations is a more important determinant of auditory distraction than the degree to which a distractor sequence contains local auditory changes.

## Introduction

It is well established that auditory distractors that deviate from the acoustic environment capture attention [1], [2], [3]. Novel auditory stimuli [1], [4] or those that deviate from prior stimulation [2], [5] are often used to study the mechanisms of auditory distraction. In the cross-modal oddball paradigm [1], [5] participants are usually required to concentrate on a simple visual classification task while task-irrelevant auditory distractors are played. Many studies have found that irregular or deviant stimuli capture more attention than standard stimuli [2], [6]. Often, irregular stimuli are simply defined as stimuli that deviate from a repetitive sequence of a specific number of identical standard stimuli [7] or as occurring with a low probability [8], [9]. Moreover, it has been suggested that auditory capture is determined by the degree to which the deviant stimulus is acoustically different from the standard stimulus [10], [11]. The detection of an auditory deviance is associated with a specific event-related potential correlate, the mismatch negativity (MMN; for an overview see [12], [13]). The concept of deviance is also central to working memory models such as Näätänen's model of attention and automaticity in audition [8], which are based on the concept of the orienting reaction [14], [15]. In simplified terms, it is assumed that incoming information is compared to a neural model of the recent auditory past, and the detection of a significant difference between the incoming and previous stimulation leads to further processing, and eventually results in a full attention switch to the auditory modality (see [16] for a similar model of attentional capture).

Deviance-based distraction can be defined in different ways. Nöstl, Marsh, and Sörqvist [17] distinguished between deviance-based distraction in terms of a local change and deviance-based distraction in terms of general expectation violation. A local change of varying degree may result when a stimulus deviates from previous stimulation. For instance, the elicitation of the MMN usually depends on an auditory stimulus deviating from a previous repetition of a standard sound [7], and an orienting response is thought to result from “a mental process in which the auditory properties of the incoming sound are compared with a memory representation of the previous sounds and are found to differ from it” (p. 909). The size of the attentional capture by the deviant may thus be related to the quality of the representation of the standard which increases as a function of the number of previous repetitions of the standard stimulus [7] and, by implication, of the rareness of the auditory deviant [1], [18], and it may be related to the size of the deviance, that is, to the degree to which the irregular auditory stimulus differs acoustically from the standard stimulus [10], [11]. In essence, the local change account of distraction predicts that any sound captures attention if it differs perceptually from previous stimuli. According to an alternative, expectation-based account [5], [19], [20], [21] auditory distraction is a function of the degree to which these stimuli violate expectations (i.e., the degree to which the acoustic input mismatches regularities that can be extracted from prior stimulation). In this case, the stimuli do not have to be rare, and their perceptual properties do not have to differ grossly from previous stimulation as long as they violate an abstract rule structure of the auditory stream. Interestingly, such irregularities may also elicit an MMN (see [22]).

The present experiments were designed to address the question of whether auditory distraction is a function of the number of local changes in the auditory distractor sequences, or whether it rather depends on the degree to which the distractor sequence violates participants' expectations about the continuation of the auditory stimulation. Note that the expectation violation account can be further subdivided by distinguishing between local violations of expectations (deviants that are inconsistent with a rule that is inherent in the immediately preceding auditory events) and global violations of expectations (deviants that are inconsistent with abstract rules that are part of the participants' long term knowledge such as the knowledge about the good continuation of sentences and melodies). Therefore a further aim of the present study is to examine the relative importance of local auditory changes, and local and global violations of expectations in determining auditory distraction.

Behavioral distraction was measured using the irrelevant sound paradigm [23]. In this paradigm, participants are required to serially recall lists of digits, letters, or words, which are visually presented. During the encoding of the target items, or during a short retention interval, auditory distractors are presented that are completely task-irrelevant, and should be ignored. In the past it has been considered a fact that the degree to which the auditory distractors contain local changes is the primary determinant of auditory distraction in this paradigm [24], [25], [26]. Whereas sequences composed of different distractors (changing state sequences) are known to produce a prominent irrelevant sound effect, repetitive sequences (steady state sequences) often fail to do so. A distractor sequence made up of different consonants such as “F J R B L K Q M”, for instance, typically impairs immediate recall for lists of digits as compared to a quiet control condition, while a single repeated consonant sequence such as “R R R R R R R R” has little or no effect (e.g., [27], [28]). This pattern of results has been replicated frequently with various types of materials [24], [25], [26], [29]. Recently, it has been established that a single deviation from a previous sequence of events (such as a change in distractor identity, inter-stimulus-interval, or voice) suffices to produce a pronounced increase in interference [30], [31], [32], [33]. Most of these manipulations implied a major local change from a previously regular auditory sequence.

However, there is also preliminary evidence that the violation of expectations may play a more important role in determining interference than the degree to which the deviant stimulus introduces local changes [17], [30]. In these studies, however, only very simple and artificial stimuli such as sequences of alternating sine tones were used. Furthermore, only the distraction of a single irregular deviant event was investigated. In the present study, we aimed at extending these findings in several ways. First, we examined whether the expectation violation account holds for more complex and naturalistic stimuli such as piano melodies (Experiment 1) and spoken texts (Experiments 2, 3, and 4). Second, we examined whether the expectation violation effect is confined to encoding, or whether it is due to a disruption of rehearsal (Experiment 3). Third, we followed Nöstl et al. [17] in contrasting directly the expectation violation account with the local changes account, but we used much stronger manipulations of the amount of local changes and expectation violation by presenting melodies and spoken texts that ended with unexpected repetitions of single tones or words, respectively. These steady state endings contained much less local changes (essentially none) than the regular endings, but at the same time grossly violated participants' expectations about the continuation of the auditory sequences. Fourth, and finally, we opted for a more general investigation of the disruptive effects of auditory deviants and examined the role of local versus global expectation violations by manipulating the preexposure to the specific auditory distractors across experiments.

## Experiment 1

### Method

#### Ethics Statement

The study was approved by the ethics committee of the medical faculty of Heinrich Heine University Düsseldorf. Participants signed an informed consent before participating in the experiment.

#### Participants

A total of 98 students (70 women) at Heinrich Heine University Düsseldorf were paid for participating or received course credit. Their ages ranged from 19 to 39 years (M = 24). All participants reported normal hearing and normal or corrected to normal vision.

#### Materials

For each trial eight to-be-remembered items were sampled randomly without replacement from the set {1, 2, … 9}. The numbers were presented consecutively at a rate of 1 per second (80 ms on, 20 ms off) in 72 point equidistant Monaco font on a white background in the centre of the 19″ computer screen. From a viewing distance of 45 cm they subtended a vertical visual angle of 1.49° and a horizontal angle of 0.92°.

**Figure 1:**
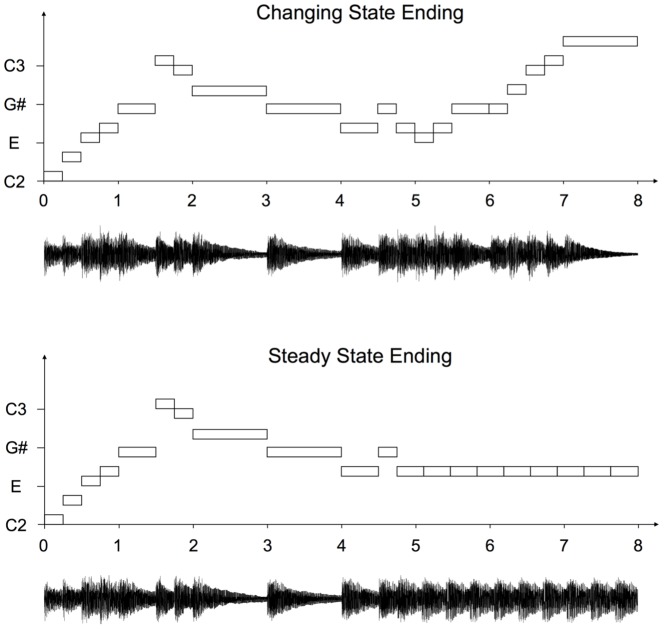
Melodies used in Experiment 1. A schematic representation of the two types of melodies used in Experiment 1 plotted against time in seconds. Every tone is represented by a rectangle. The length of the tone is indicated by the width of the rectangle, the base frequency by its vertical position. The total number of tones per sequence is identical in both conditions.

Auditory distractor sequences were eight piano melodies in the key of C major that were generated using Apple's GarageBand music editing software. The melodies lasted 8 s and were normalized to minimize amplitude differences amongst the stimuli. Each melody was repeated three times during a passive listening period. During the subsequent visual presentation of the to-be-remembered items, the melody was either repeated a fourth time in the changing state ending condition, or, in the steady state ending condition, began as before, but in its third measure a sequence of a repeated single tone replaced the regular ending. The length of this repeated tone corresponded to the average length of the tones in the corresponding section of the changing state ending. Hence, melodies with a changing state ending and those with a steady state ending contained the same number of auditory events (see Figure 1). Also note that the frequency and amplitude spectrum of the repeated tone of the steady state ending were always identical to the frequency and amplitude spectrum of the last regular tone of each melody so that there was no abrupt change in the acoustic properties associated with a steady state ending.

#### Procedure

Throughout the experiment participants wore headphones with high-insulation hearing protection covers that were plugged directly into the Apple iMac computer that controlled the experiment. All sounds were presented binaurally at about 65 dB(A). Standard written instructions on the computer screen informed participants to ignore any sound and to avoid pronouncing the to-be-remembered items.

Participants completed 40 trials, which were divided into two blocks. The training block (16 trials) consisted of eight quiet and eight changing state ending trials. The experimental block (24 trials) consisted of eight trials in each of the three auditory distractor conditions (quiet, changing state ending, steady state ending). The eight melodies were presented once in each block and condition. Within blocks trials were presented in random order. The training block enabled the participants to familiarize with the task and to build up expectations about the continuation of the melodies.

Each trial began with a passive listening period of 24 s during which the sampled melody was presented three times consecutively. During the subsequent visual presentation of the to-be-remembered list of eight numbers, the melody was either repeated a fourth time (changing state ending condition), or began as before, but in its third measure a repeated single tone replaced the regular ending (steady state ending condition). In the quiet condition silence was played during list presentation.

Immediately after each trial, participants were required to recall the visually presented items in the order in which they had been presented. A series of eight question marks, one for each of the serial positions, prompted forward serial recall. Participants used the keyboard's number pad to enter the items in the order in which they had been presented, with each number replacing one question mark. Participants could omit a serial position by pressing a “don't know” button on the keyboard, as a consequence of which a hyphen replaced the question mark. As in many irrelevant sound experiments, including our own (e.g., [34], [35], [36], [37]), participants were allowed to correct their responses, but were nevertheless required to recall the items in forward order. Recall was self-paced and terminated by pressing the space bar (provided the last question mark had been replaced). Performance feedback was given after each trial.

The experiment took approximately 30 min to complete, after which participants were offered an explanation as to the purpose of the experiment.

#### Design

A repeated measures design was used with auditory distractor condition (quiet, changing state ending, steady state ending) and serial position (1–8) as the independent variables and serial recall performance as the dependent variable (remembered numbers were scored as correct when they were reproduced in the serial position in which they had been presented). Of primary interest was the comparison between the changing state ending condition and the steady state ending condition. Given α = β = .05, the assumption that the population correlation between the levels of the repeated measures factor is ρ = .5, and a sample size of N = 98, an effect of size f = 0.18 could be detected. The power calculation was conducted using G*Power [38].

A multivariate approach was used for all within-subject comparisons. In our application, all multivariate test criteria correspond to the same (exact) F statistic, which is reported. The level of α was set to .05 for all analyses. Partial η^2^ is reported as a measure of the sample effect size.

### Results

**Figure 2:**
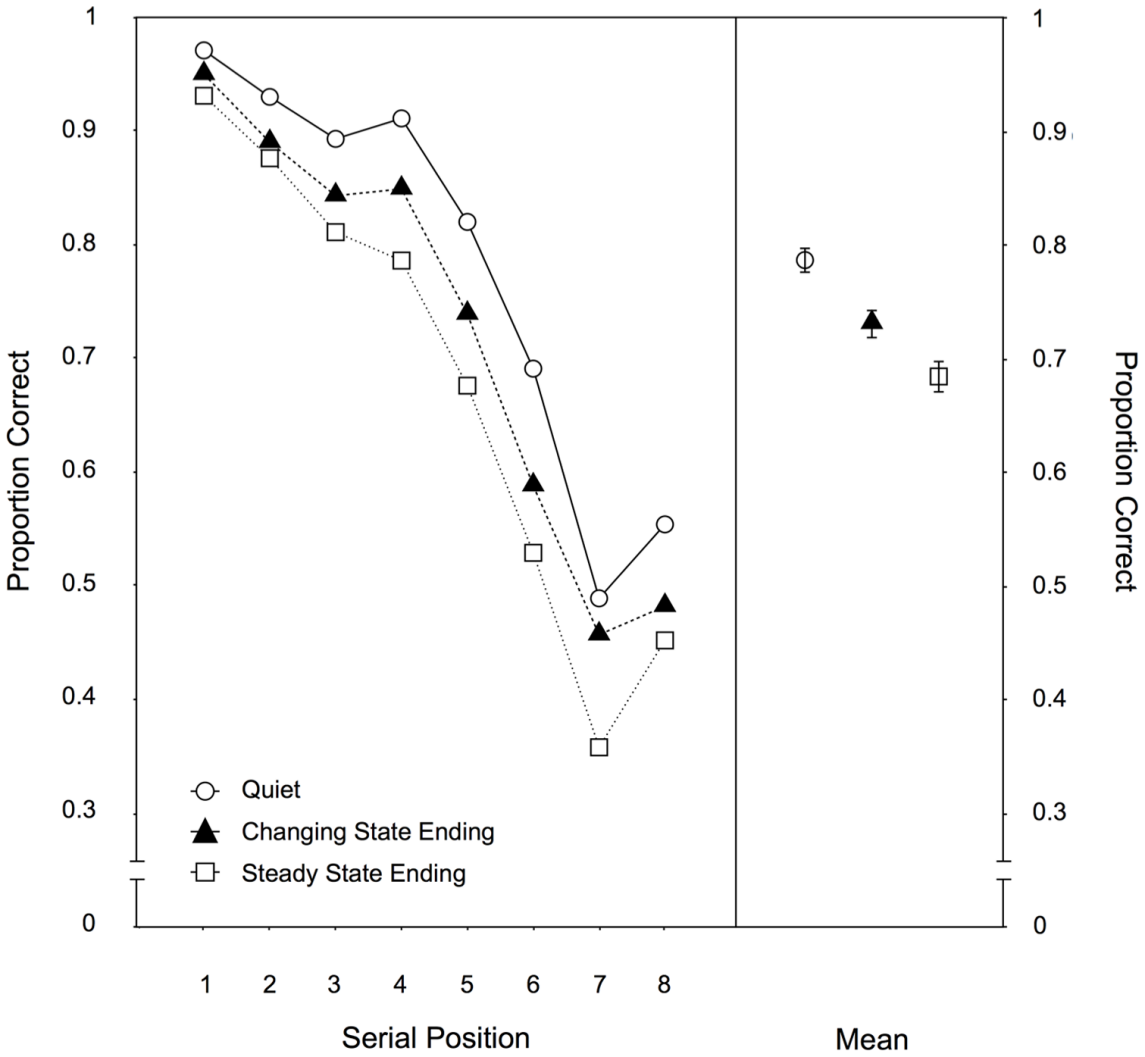
Serial position data for Experiment 1. Recall performance as a function of auditory distractor condition (quiet, changing state ending, steady state ending) for each serial position (left panel) and averaged across positions (right panel) in Experiment 1. The error bars depict the standard errors of the means.

Serial position. Figure 2 illustrates the serial recall performance as a function of auditory distractor condition across the eight serial positions. A 3×8-MANOVA yielded significant main effects of the auditory distractor condition, F(2,96) = 38.80, p<.001, 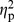  = .45, and serial position variables, F(7,91) = 63.85, p<.001, 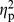  = .83. The interaction of both variables was also significant, F(14,84) = 3.42, p<.001, 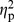  = .36. Orthogonal contrasts showed that the typical irrelevant sound effect could be observed in that recall performance was reduced in the distractor conditions relative to the quiet condition, F(1,97) = 68.14, p<.001, 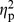  = .41. Interestingly, melodies with an unexpected steady state ending interfered more with serial recall than melodies with the expected changing state ending, F(1,97) = 16.17, p<.001, 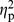  = .14.

**Figure 3:**
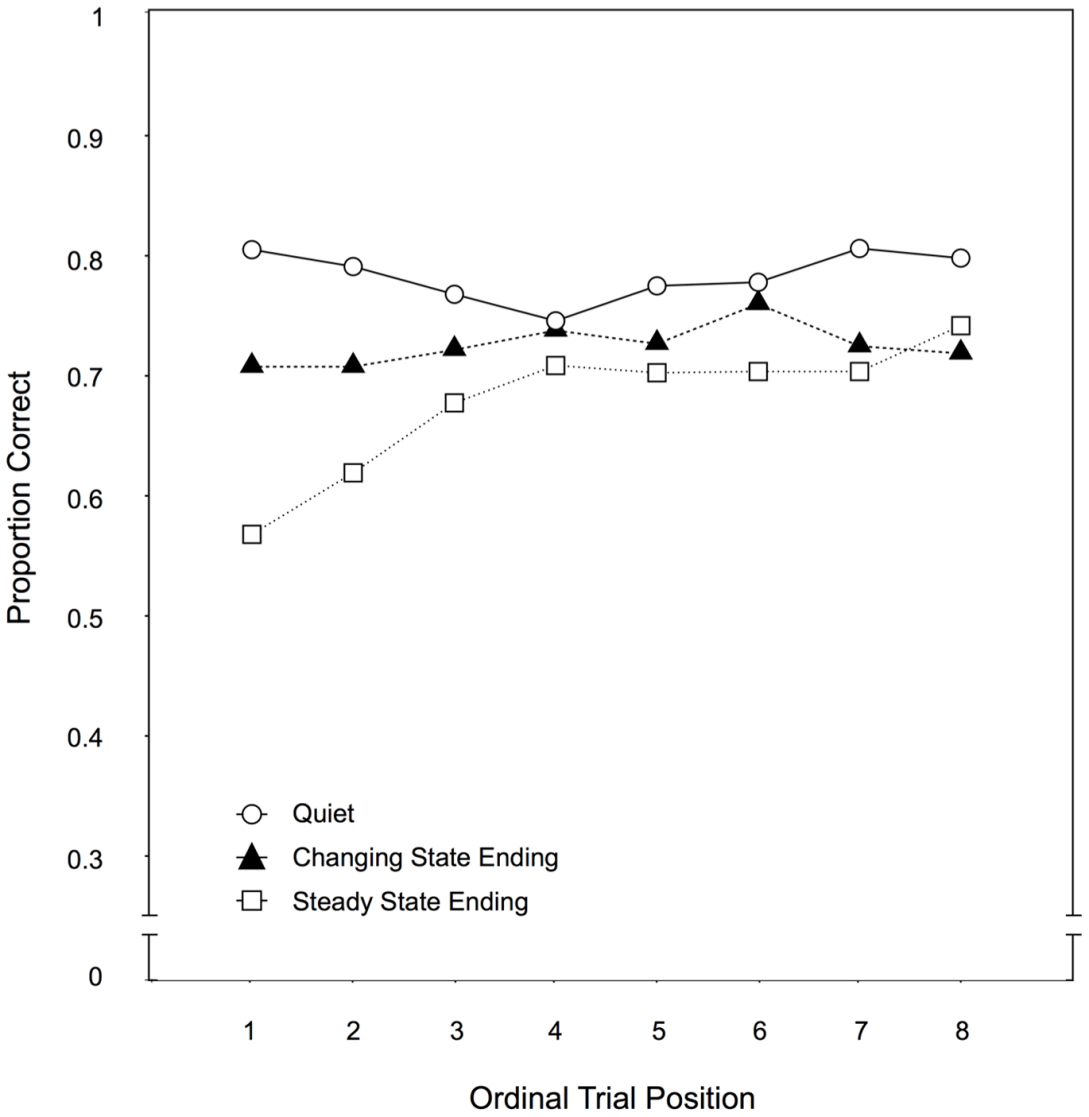
Across-trial performance for Experiment 1. Recall performance as a function of the auditory distractor condition (quiet, changing state ending, steady state ending) and the ordinal position of a trial within a condition in Experiment 1.

Across-trial performance. By plotting the recall performance for each distractor condition as a function of the ordinal position of the eight trials within a condition (see Figure 3), it can be illustrated to what extent participants improved over the course of the experiment. Specifically, a progressively smaller gap in recall performance between the steady state ending condition and the quiet condition would indicate that habituation had occurred to the steady state endings. When these two conditions were compared, there were significant main effects of auditory distractor condition, F(1,97) = 76.84, p<.001, 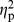  = .44, and of ordinal position, F(7,91) = 4.00, p = .001, 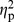  = .24. Most importantly, the interaction of both variables was also significant, F(7,91) = 7.30, p<.001, 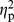  = .24. In contrast, when recall performance in the changing state ending condition was compared to the quiet condition, there was also a main effect of auditory distractor condition, F(1,97) = 27.58, p<.001, 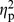  = .22, but neither a main effect of ordinal position, F(7,91) = 0.60, p = .758, 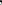  = .04, nor an interaction of both variables, F(7,91) = 1.49, p = .182, 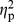  = .10.

### Discussion

Immediate serial recall was disrupted by task-irrelevant piano melodies. Most interestingly, melodies, which unexpectedly turned into steady state sequences had a particularly high disruptive potential despite being acoustically much less variable than the regular melodies. This is a surprising result because the extent to which the serial recall performance is impaired typically decreases when the irrelevant sound becomes less variable [24], [27], [39]. Although it has occasionally been demonstrated that a single unexpected repetition of a standard tone may capture attention (e.g., [30]), the present study is the first showing that replacing half of the local changes of a changing state sequence by an unexpected non-changing steady state sequence of repetitive tones can be more disruptive than an entire changing state sequence with the full set of local changes. Note that this effect cannot have been caused by a particularly abrupt change in amplitude or frequency at the transition from the melody to the steady state ending, because the last regular tone was always identical to the repeating tones in terms of frequency and amplitude spectrum. Another interesting aspect is that the disruptive effect of melodies with a steady state ending decreased after repeated exposure to such endings while that of melodies with a changing state ending remained constant across trials. The performance drop caused by the unexpected ending was most prominent on the first occasion and decreased continuously over the course of the experiment.

The present results shed light on the determinants of attentional capture. First, and consistent with recent studies [5], [17], [33], [40], auditory distraction was more strongly affected by expectation violation than by the number of local changes in the to-be-ignored stream. The results are inconsistent with the local change account of auditory distraction, according to which a sound's disruptive potential is determined solely by the difference between each distractor item and the preceding auditory stimulation. Instead, the results support an expectation-based account, according to which the auditory system predicts the occurrence of tones based on a set of rules even when the sounds are ignored (a similar mechanism may exist in vision, see [41]), and auditory distraction is a function of the degree to which a sound violates these predictive rules. Apparently, it seems that the auditory system uses rather complex neural models (e.g., whole melodies) to predict the continuation of a given distractor sequence. Note that these expectations may be either global and unspecific (the steady state endings violated participants' knowledge about the continuation of melodies in general) or local and specific (each melody was played three times before each trial, which should have allowed for the development of a neural model of the specific melody). However, finding that habituation occurred to the steady state endings although in each trial a different melody was used suggests that a neural model was built up that comprised rather stimulus-unspecific regularities. Habituation did not occur to a specific melody with a steady state ending, but to the occurrence of steady state endings as such. This is consistent with previous observations [33], [36] showing that habituation can occur to abstract regularities of distractor sequences.

## Experiment 2

The first aim of Experiment 2 was to replicate conceptually the results of Experiment 1. The second aim was to examine whether the expectation violation effect can be replicated using another type of highly complex, naturalistic distractor material (i.e., distractor speech). In the auditory distraction literature, it is a recurrent issue of controversy whether the disruption by irrelevant speech is governed by the same principles as the disruption by non-speech sounds [42], [43]. Therefore, it is interesting to examine whether it is possible to replicate the findings of Experiment 1 with speech distractors.

### Method

#### Participants

A total of 59 students (44 women) at Heinrich Heine University Düsseldorf were paid for participating or received course credit. Their ages ranged from 19 to 47 years (M = 24). All participants reported normal hearing and normal or corrected to normal vision.

#### Materials, Procedure, and Design

**Figure 4:**
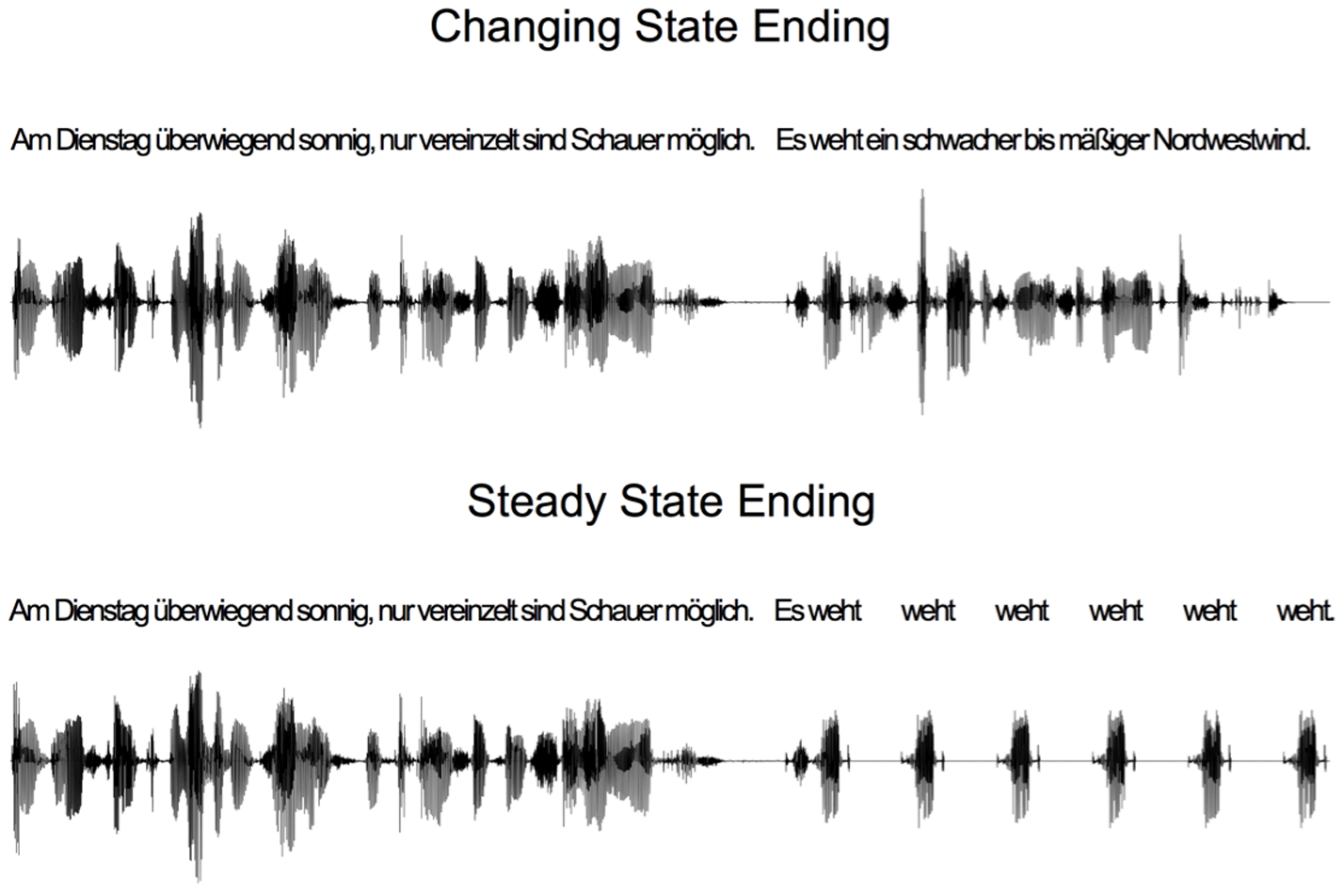
Sentences used in Experiment 2. A schematic representation of the two types of sentences used in Experiment 2. The translations read “Tuesday mostly sunny with scattered showers. It blows a weak to moderate northeasterly wind.” and “Tuesday mostly sunny with scattered showers. It blows blows blows blows blows blows.”, respectively.

Materials, procedure, and design were identical to those of Experiment 1 with the following exceptions. Auditory distractors were sentences taken from Bell et al. [44]. The sentences were sampled from eight different categories (weather forecast, prose text, cooking recipe, scientific textbook, poem, operating manual, road message, aphorism) and were spoken by the same male voice. Each distractor sentence lasted for 8 s. Each sentence was repeated three times during a passive listening period. During the visual presentation of the item list, the sentence was either repeated a fourth time (changing state ending condition), or a repeated monosyllabic word replaced the regular ending (steady state ending condition). The number of repetitions within the steady state ending corresponded to the number of words in the regular ending (see Figure 4 for an exemplary sentence). All sounds were presented binaurally at about 63 dB(A).

Given a sample size of N = 59, an effect of size f = 0.24 could be detected between the changing state ending condition and the steady state ending condition.

### Results

**Figure 5:**
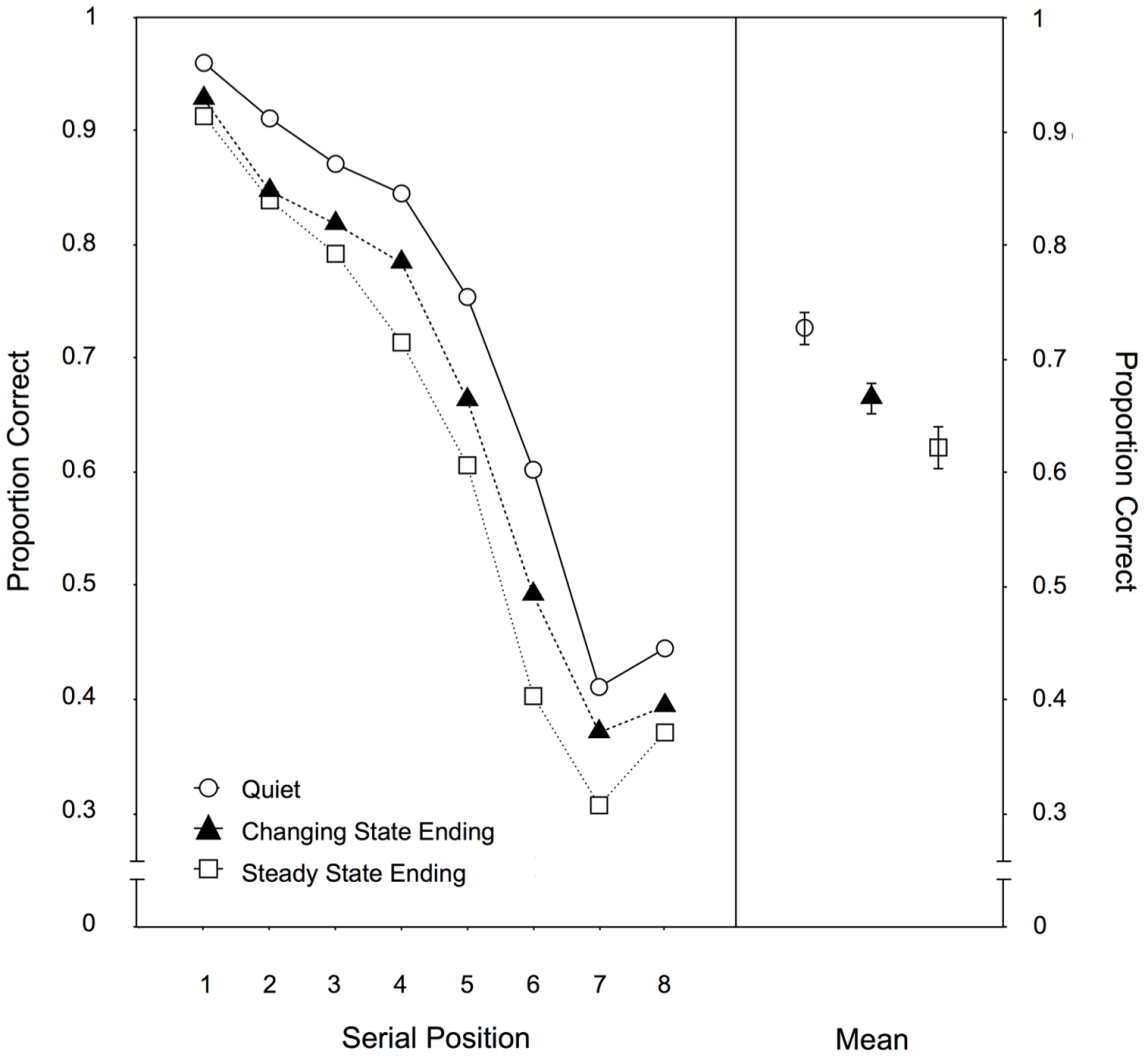
Serial position data for Experiment 2. Recall performance as a function of auditory distractor condition (quiet, changing state ending, steady state ending) for each serial position (left panel) and averaged across positions (right panel) in Experiment 2. The error bars depict the standard errors of the means.

Serial position. Figure 5 shows the serial recall performance as a function of auditory distractor condition across the eight serial positions. A 3×8-MANOVA yielded significant main effects of the auditory distractor condition, F(2,57) = 33.22, p<.001, 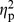  = .54, and serial position variables, F(7,52) = 38.23, p<.001, 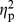  = .84. The interaction of both variables was not significant, F(14,45) = 1.65, p = .102, 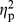  = .34. Orthogonal contrasts revealed the typical irrelevant sound effect on serial recall performance: More errors were made in the distractor conditions relative to the quiet condition, F(1,58) = 56.27, p<.001, 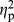  = .49. Parallel to the melodies with the steady state ending in Experiment 1, sentences that ended unexpectedly with a repeated word interfered more with serial recall than sentences with the expected changing state ending, F(1,58) = 11.95, p = .001, 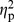  = .17.

**Figure 6:**
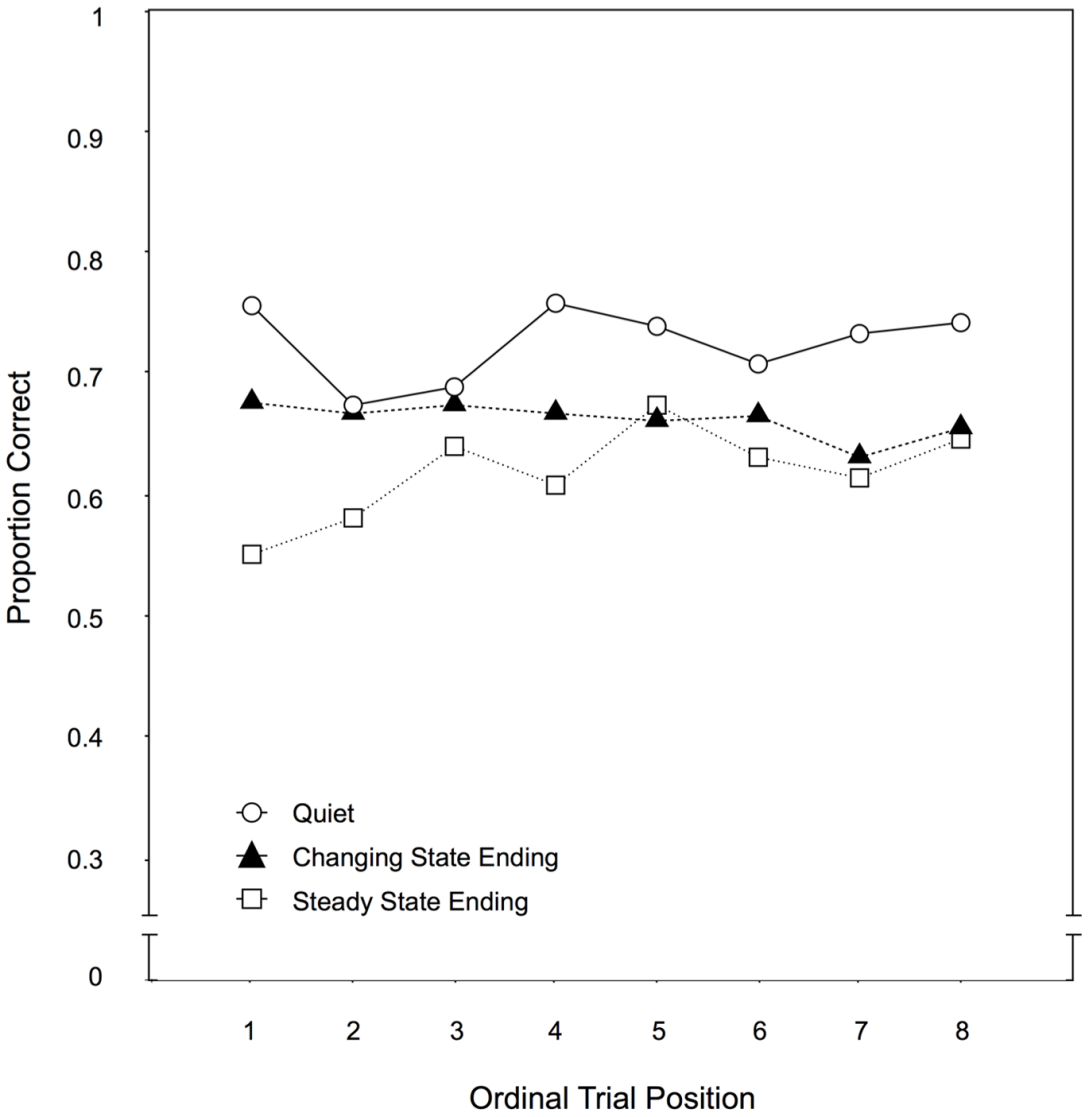
Across-trial performance for Experiment 2. Recall performance as a function of the auditory distractor condition (quiet, changing state ending, steady state ending) and the ordinal position of a trial within a condition in Experiment 2.

Across-trial performance. Figure 6 illustrates the serial recall performance as a function of the ordinal position of the eight trials. When the steady state ending condition and the quiet condition were compared, there was a main effect of the auditory distractor condition variable, F(1,58) = 67.07, p<.001, 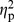  = .54. Ordinal position had no effect, F(7,52) = 1.46, p = .203, 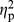  = .16. The interaction of both variables also failed to reach statistical significance, F(7,52) = 1.99, p = .075, 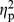  = .21, but there was a descriptive trend towards a recovery of performance in the steady state ending condition. When the changing state ending condition was compared to the quiet condition, there was a main effect of auditory distractor condition, F(1,58) = 23.69, p<.001, 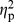  = .29, but neither a main effect of ordinal position, F(7,52) = 0.73, p = .645, 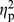  = .09, nor an interaction of both variables, F(7,52) = 1.33, p = .254, 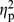  = .15.

### Discussion

Experiment 2 further validates the finding that distractor sequences, which unexpectedly turn into a steady state sequence may produce greater disruption than acoustically more variable sequences that are presented in line with expectations. Thus, the disruptive effect of the unexpected steady state sequences is a more general effect that is not limited to non-verbal distractor material. As in Experiment 1, disruption caused by the unexpected distractor repetitions decreased with repeated presentation. From the first to the eighth trial participants improved markedly in the steady state ending condition (10%), but not in the changing state ending (−2%) and quiet conditions (−2%). Note, however, that the interaction between ordinal position and the auditory distractor variable failed to reach significance, so that this descriptive trend should only be interpreted cautiously.

## Experiment 3

Experiment 3 examines whether the disruptive effect of unexpected steady state endings is confined to encoding, or is also found when the unexpected distractor repetitions are presented during a retention interval after encoding has been completed. Examining this question is relevant, because it has been recently argued that the effect of attentional capture by deviant stimuli only disrupts the encoding of the target items, but does not affect the maintenance of the items in working memory [31]. Although specific effects of attentional capture on encoding seem possible given that encoding of visual items involves other (perceptual) processes than maintaining these items in working memory, there are also a number of overlapping mechanisms involved in the encoding and the rehearsal of verbal material, and it has been previously found that the disruptive effect of irrelevant sound on serial recall is identical regardless of whether the auditory distractors are played during encoding or during a short retention interval [45], [46].

To this end, an encoding group was contrasted with a retention group in Experiment 3. In the encoding group, sentences with a changing state ending and those with a steady state ending differed from each other during the presentation of the item list, just like in Experiments 1 and 2. In the retention group, both types of sentences were identical during list presentation and differed from each other during a retention interval. In other words, the unexpected distractor repetitions only occurred after the presentation of the visual items was complete and the retention interval had already begun. If the unexpected steady state endings only interfere with the encoding of the item list, then a greater disruption compared to the changing state endings should only be observed in the encoding group, but not in the retention group. If, by contrast, the unexpected steady state endings interfere with the maintenance of the items in working memory, then a greater disruption compared to the changing state endings should be observed in both groups.

### Method

#### Participants

A total of 69 students (44 women) at Heinrich Heine University Düsseldorf were paid for participating or received course credit. Their ages ranged from 18 to 55 years (M = 24). All participants reported normal hearing and normal or corrected to normal vision. Random group assignments led to n = 35 in the encoding group and n = 34 in the retention group.

#### Materials, Procedure, and Design

**Figure 7:**
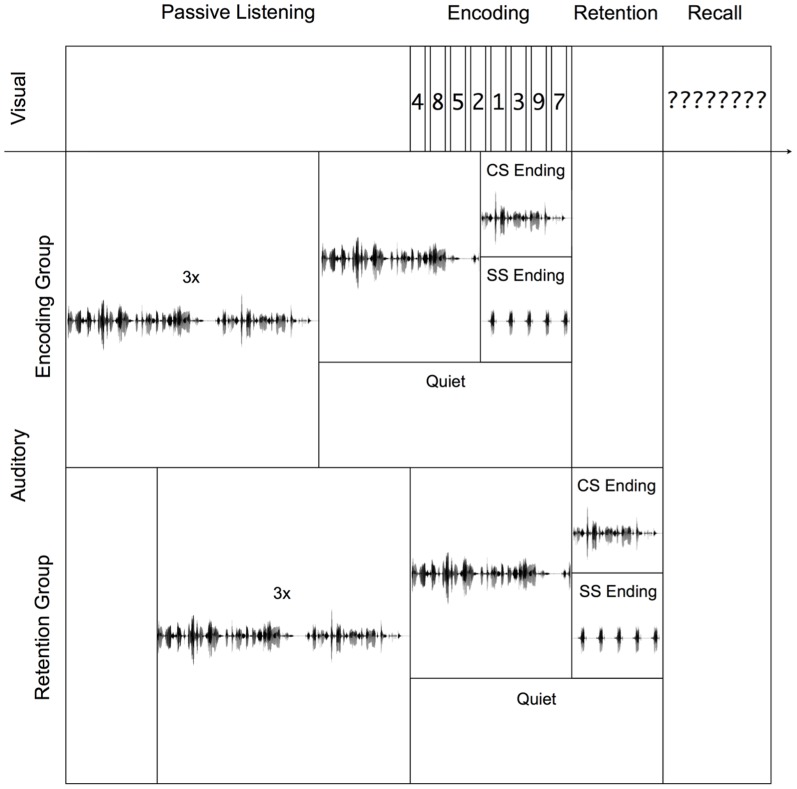
Stimulus presentation in Experiment 3. Illustration of an exemplary trial in Experiment 3 for both experimental groups. In the encoding group sentences with a changing state ending and those with a steady state ending differ from each other during the presentation of the item list, while in the retention group they differ from each other in a retention interval after encoding is completed.

Materials, procedure, and design were identical to those of Experiment 2 with the following exceptions. The to-be remembered items were presented at a quicker rate (500 ms on, 125 ms off) than in Experiment 1, and a retention interval of 3 seconds was inserted between the offset of the last to-be remembered number and the recall phase. Participants were assigned to two groups. In the encoding group, the unexpected steady state ending (or the corresponding changing state ending) occurred during list presentation, as in Experiments 1 and 2. In the retention group, the unexpected steady state ending (or the corresponding changing state ending) occurred during the retention interval (see Figure 7). It is important to note the regular part of the sentence (e.g., “Am Dienstag überwiegend sonnig, nur vereinzelt sind Schauer möglich. Es weht”; see also Figure 4) occurred during list presentation. The expectation violation did not occur until the first repetition of the final word of that sentence (e.g., “Am Dienstag überwiegend sonnig, nur vereinzelt sind Schauer möglich. Es weht weht weht…”; see also Figure 4). The first repetition of that final word and thus the expectation violation occurred after the retention interval had already begun.

Participants were randomly assigned to one of the two groups (presentation group, retention group) prior to the experiment, resulting in a 2 (group) by 3 (distractor condition) by 8 (serial position) design. Given a sample size of N = 69, an effect of size f = 0.22 could be detected between the changing state ending condition and the steady state ending condition.

### Results

**Figure 8:**
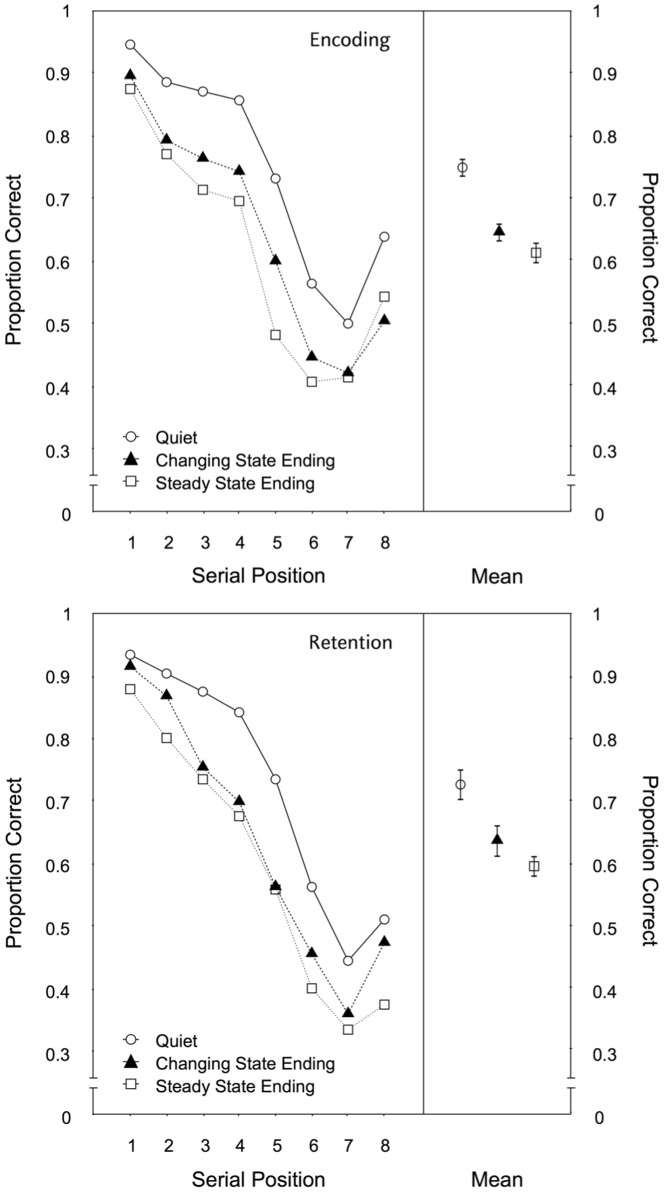
Serial position data for Experiment 3. Recall performance as a function of auditory distractor condition (quiet, changing state ending, steady state ending) for each serial position (left panel) and averaged across positions (right panel) in Experiment 3. In the upper panel the recall performance for the encoding group is shown, in the lower panel that for the retention group. The error bars depict the standard errors of the means.

Serial position. Figure 8 illustrates the serial recall performance for both groups as a function of auditory distractor condition across the eight serial positions. A 2×3×8-MANOVA yielded no main effect of group, F(1,67) = 0.23, p = .631, 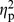 <.01, but significant main effects of the auditory distractor condition, F(2,66) = 52.13, p<.001, 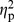  = .61, and serial position variables, F(7,61) = 26.99, p<.001, 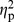  = .76. The interaction of auditory distractor condition and serial position was also significant, F(14,54) = 2.44, p = .010, 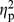  = .39. Most importantly, the auditory distractor manipulation had identical effects regardless of whether the unexpected steady state or expected changing state endings occurred during encoding or retention, F(2,66) = 0.139, p = .870, 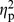 <.01. Finally, there was neither an interaction of presentation stage with serial position, F(7,61) = 1.62, p = .147, 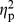  = .16, nor a three-way-interaction, F(14,54) = 1.68, p = .087, 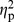  = .30.

When the data for both groups were analyzed separately, the steady state endings were more disruptive than the changing state endings in both the encoding group, F(1,34) = 5.01, p = .032, 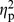  = .13, and the retention group, F(1,33) = 5.98, p = .020, 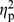  = .15, confirming that unexpected changes within a distractor sequence disrupt the maintenance of the to-be-remembered items and not only their encoding. Note that even the size of the effect is very similar in both groups.

**Figure 9:**
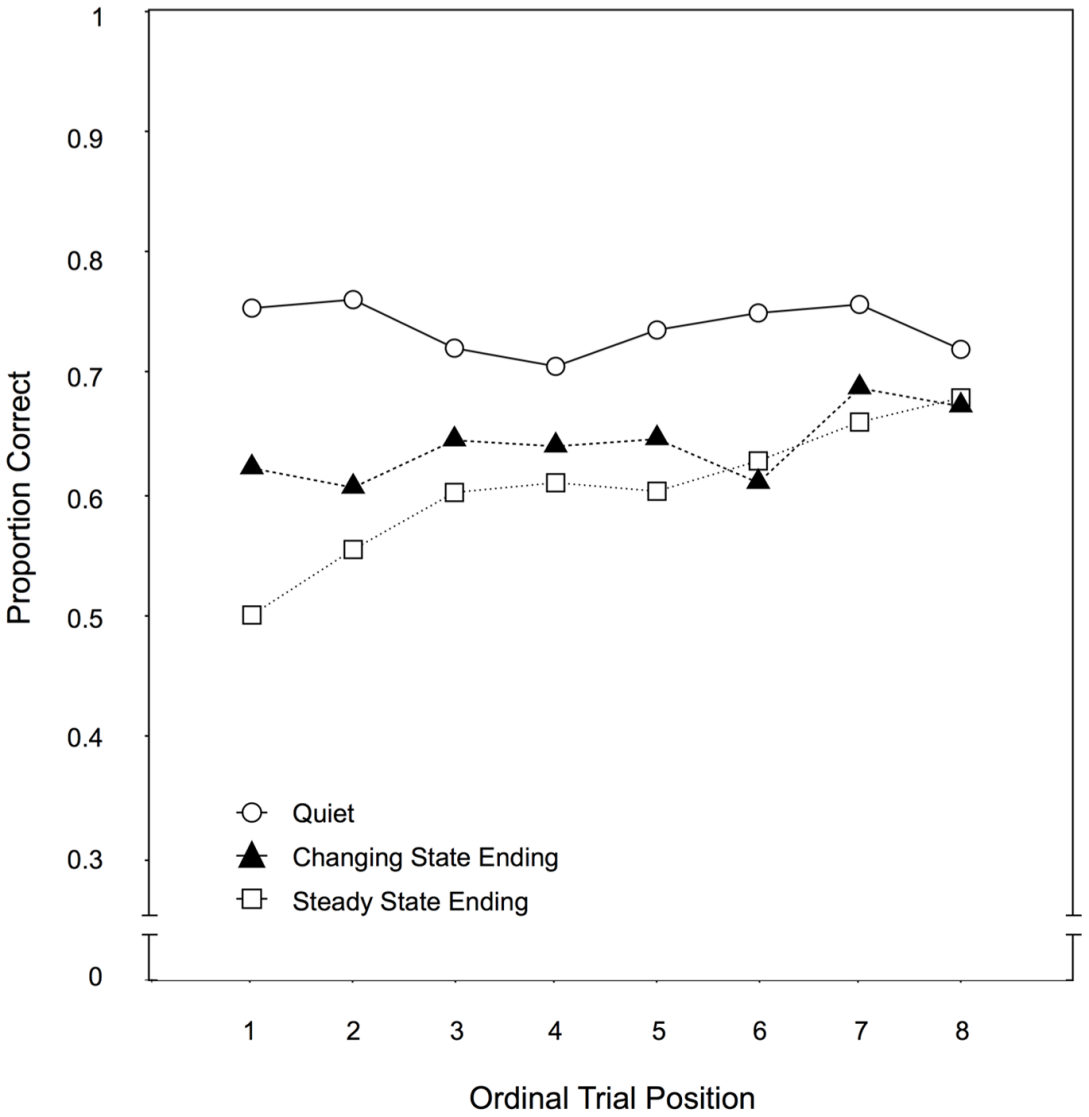
Across-trial performance for Experiment 3. Recall performance as a function of the auditory distractor condition (quiet, changing state ending, steady state ending) and the ordinal position of a trial within a condition in Experiment 3 for both experimental groups combined.

Across-trial performance. Figure 9 illustrates the serial recall performance as a function of the ordinal position for both experimental groups combined. When the steady state ending condition and the quiet condition were compared there were main effects of auditory distractor condition, F(1,67) = 104.83, p<.001, 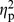  = .61 [encoding group: F(1,34)  = 45.56, p<.001, 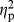  = .57; retention group: F(1,33)  = 67.78, p<.001, 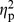  = .66] and ordinal position, F(7,61)  = 2.87, p = .012, 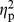  = .25 [encoding group: F(7,28)  = 4.95, p = .001, 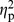  = .55; retention group: F(7,27)  = 0.99, p = .456, 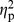  = .21]. Critically, the interaction of both variables was also significant, F(7,61)  = 4.19, p = .001, 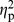  = .33 [encoding group: F(7,28)  = 2.40, p = .047, 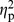  = .38; retention group: F(7,27)  = 2.82, p = .024, 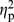  = .42], showing that habituation had occurred to the disruption by unexpected steady state endings over the course of the experiment. When the changing state condition was compared to the quiet condition, there was a main effect of auditory distractor condition, F(1,67)  = 53.78, p<.001, 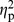  = .45 [encoding group: F(1,34)  = 26.25, p<.001, 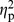  = .44; retention group: F(1,33)  = 28.50, p<.001, 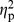  = .46]. In contrast, ordinal position had no effect on serial recall performance, F(7,61)  = 0.90, p = .511, 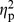  = .09 [encoding group: F(7,28)  = 1.02, p = .440, 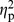  = .20; retention group: F(7,27)  = 1.32, p = .279, 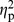  = .26]. The interaction of both variables was also nonsignificant, F(7,61)  = 1.39, p = .226, 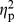  = .14 [encoding group: F(7,28)  = 0.95, p = .483, 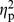  = .19; retention group: F(7,27)  = 1.99, p = .093, 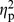  = .34]. Thus, there was no evidence for an unspecific habituation to sentences with changing state endings.

### Discussion

As in Experiments 1 and 2, sequences with an unexpected steady state ending produced greater disruption than sequences that ended in line with expectations with a changing state ending. The attentional capture effect elicited by the unexpected steady state endings was found to be independent of whether the unexpected steady state ending occurred during list presentation or during the retention interval. Experiment 3 therefore clearly shows that expectation violations do not selectively affect the encoding of the to-be-remembered items, but most notably their maintenance in working memory.

## Experiment 4

In a strict sense, the unexpected steady state endings violated two kinds of expectations. First, they violated local and specific expectations that have been built-up in the passive listening period when encountering the interrupted melody or sentence three times before each trial. Second, they violated global and unspecific expectations based on long-term knowledge about the typical continuation of melodies (Experiment 1) and sentences (Experiments 2 and 3). The goal of Experiment 4 was to examine whether local expectations are necessary, or whether violating global expectations suffices to produce an expectation violation effect. To this end the passive listening period was omitted. Thus, there was no opportunity to develop a local and specific expectation about the continuation of a sentence with steady state ending. If violations of local expectations based on a neural model of the previously presented melodies and sentences caused the attentional capture effects in Experiments 1, 2, and 3, then we should observe no evidence of disruption due to attentional capture in Experiment 4. If, in contrast, violations of global expectations suffice to produce an attentional capture effect, then the pattern of results should be similar to those in Experiments 1, 2, and 3 in that greater disruption by steady state in comparison to changing state sentences should be observed.

### Method

#### Participants

A total of 67 students (53 women) at Heinrich Heine University Düsseldorf were paid for participating or received course credit. Their ages ranged from 18 to 40 years (M = 23). All participants reported normal hearing and normal or corrected to normal vision.

#### Materials, Procedure, and Design

Materials, procedure, and design were identical to those of Experiment 2 with the following exceptions. The passive listening phase was omitted. Participants thus heard each sentence with either a changing state or a steady state ending only once. A total of 24 sentences were used, and a different sentence was selected as auditory distractor in each trial. Given a sample size of N = 67, an effect of size f = 0.22 could be detected between the changing state ending condition and the steady state ending condition.

### Results

**Figure 10:**
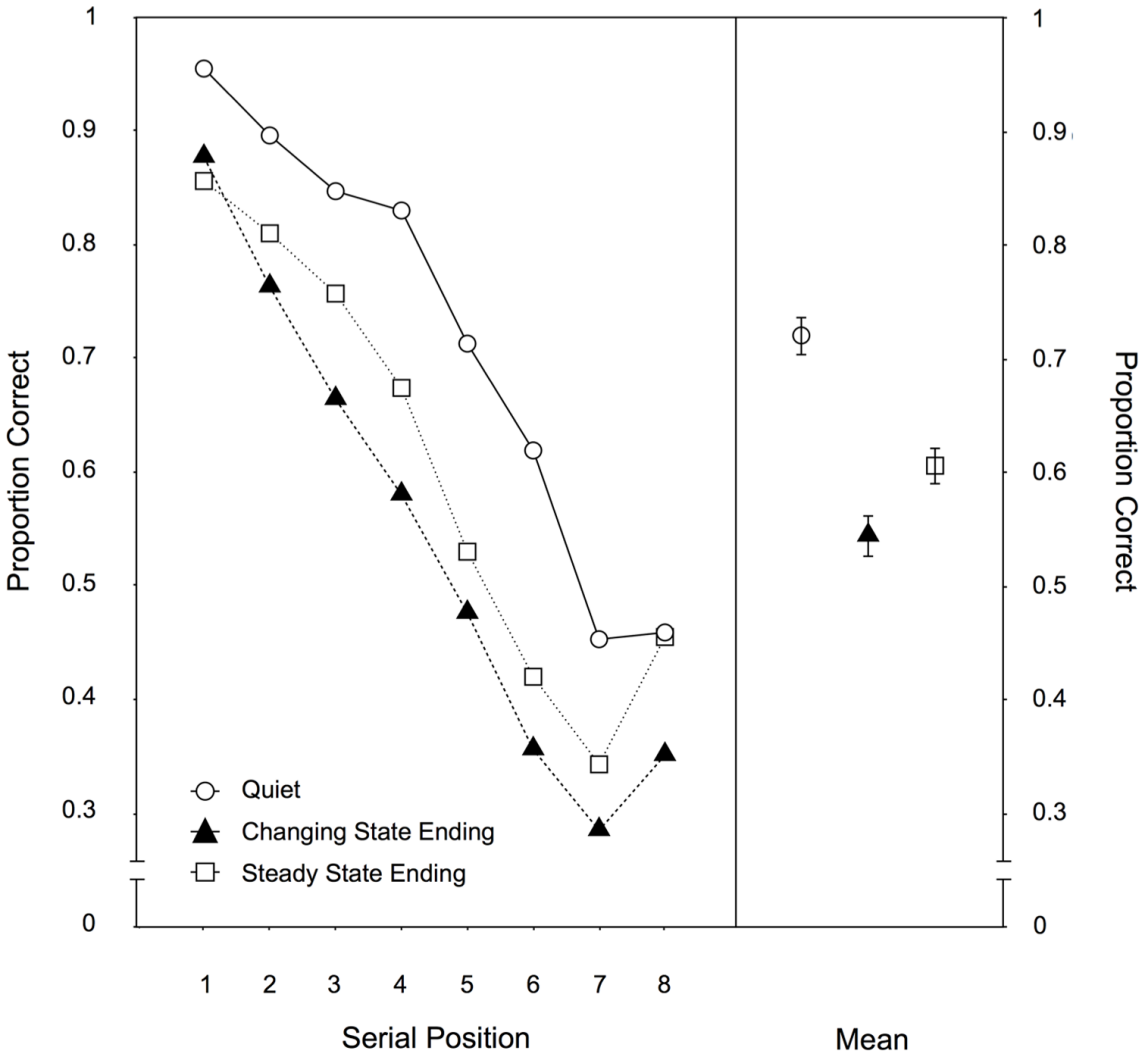
Serial position data for Experiment 4. Recall performance as a function of auditory distractor condition (quiet, changing state ending, steady state ending) for each serial position (left panel) and averaged across positions (right panel) in Experiment 4. The error bars depict the standard errors of the means.

Serial position. Figure 10 shows the serial recall performance as a function of auditory distractor condition across the eight serial positions. A 3×8-MANOVA revealed significant main effects of the auditory distractor condition, F(2,65)  = 65.40, p<.001, 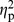  = .67, and serial position variables, F(7,60)  = 47.81, p<.001, 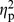  = .85. The interaction of both variables was also significant, F(14,53)  = 1.65, p<.001, 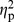  = .53. Orthogonal contrasts confirmed the irrelevant sound effect on serial recall performance, F(1,66)  = 122.04, p<.001, 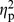  = .65. When the two types of distractor sentences were compared with each other, however, the pattern of results found in Experiments 1, 2, and 3 was reversed: When the passive listening phase was omitted, sentences that ended with a repeated word were less disruptive than sentences with a changing state ending, F(1,66)  = 16.77, p<.001, 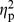  = .20.

**Figure 11:**
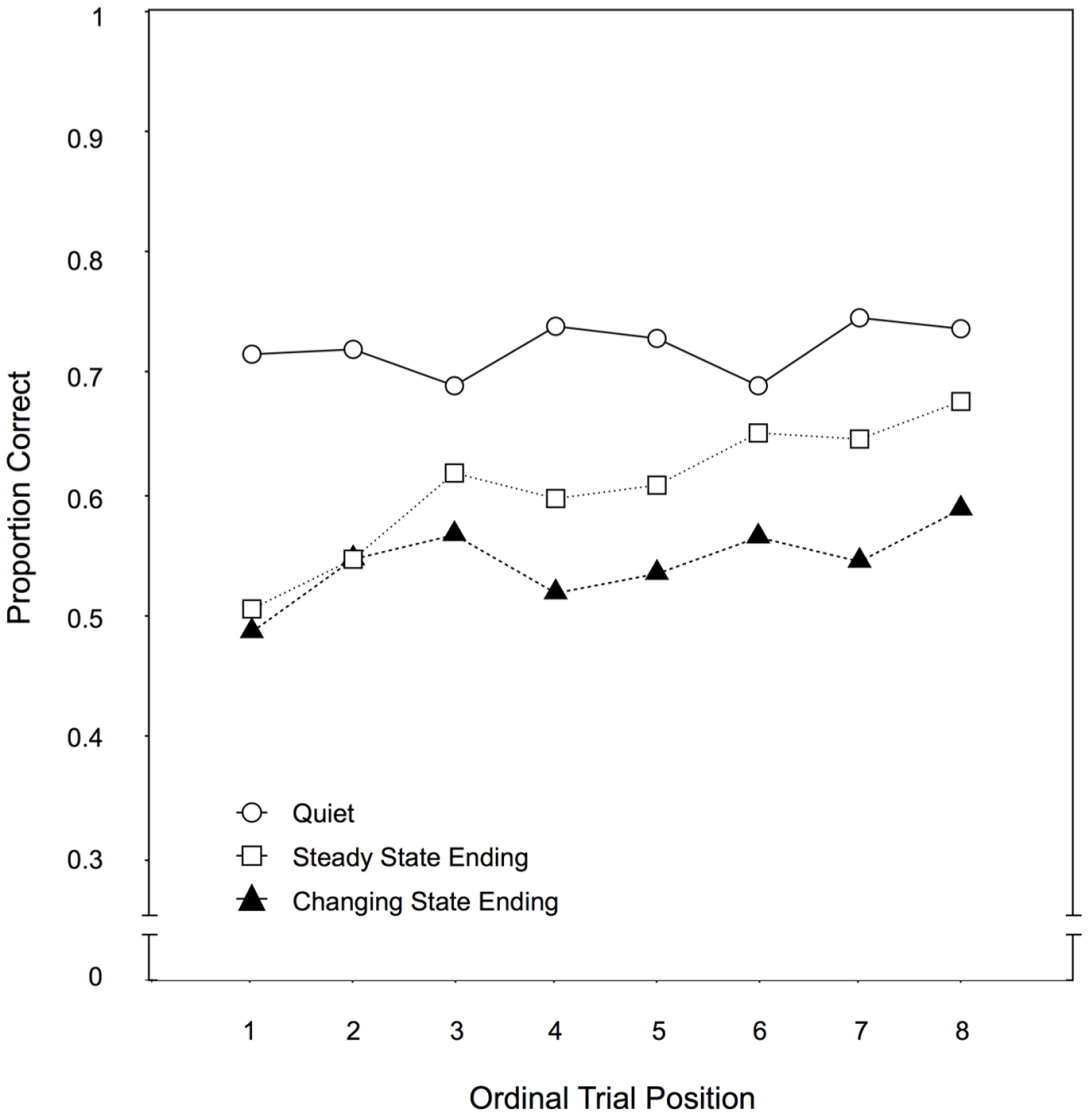
Across-trial performance for Experiment 4. Recall performance as a function of the auditory distractor condition (quiet, changing state ending, steady state ending) and the ordinal position of a trial within a condition in Experiment 4.

Across-trial performance. Figure 11 illustrates the serial recall performance as a function of the ordinal position over the course of the experiment. When the steady state ending condition and the quiet condition were compared, there were main effects of auditory distractor condition, F(1,66)  = 60.90, p<.001, 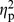  = .48, and ordinal position, F(7,60)  = 3.82, p = .002, 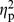  = .31. There was a significant interaction between both variables, F(7,60)  = 3.03, p = .009, 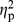  = .26, suggesting that, parallel to the previous experiments, habituation had occurred to the steady state endings. When recall performance in the changing state ending condition was compared to the quiet condition, there was a main effect of auditory distractor condition, F(1,66)  = 126.88, p<.001, 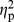  = .66, whereas ordinal position had no effect, F(7,60)  = 0.89, p = .520, 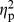  = .09. As in Experiments 1, 2, and 3, there was also no interaction of both variables, F(7,60)  = 1.62, p = .148, 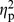  = .16.

### Discussion

In contrast to Experiments 1, 2, and 3, sequences that ended with a repeated distractor no longer caused more, but markedly less disruption than sequences with a changing state ending when there was no opportunity for participants to develop local and specific expectations about the to-be interrupted sentences. Thus, the number of local changes, that is, the acoustic variability of the distractor sequences, determined interference when no neural model existed of the specific speech sequence that was used as distractor material. This is consistent with previous observations that auditory distraction depends on a robust neural model of the standard stimulus [7], but extends these findings to much more complex distractor material. The expectation violation effect observed in Experiments 1, 2, and 3 can therefore be traced back to the violation of local expectations.

Violations of global expectations based on long-term (grammatical, syntactical, and semantic) knowledge about the typical continuation of sentences seem to play a minor role at best. Nevertheless, as in Experiments 1, 2, and 3 there was evidence for habituation to the disruptive effect of the steady state endings. This finding could be taken as evidence that the unexpected steady state ending initially elicited a small and transient attentional capture effect that was superimposed by a more pronounced effect of the number of local changes. Hence, the results of Experiment 4 suggest that interference occurs at many levels. First, in line with a bulk of literature on the irrelevant sound effect, local changes (i.e., acoustic differences between adjacent distractor items) in the distractor material cause auditory distraction. Second, there is an even more pronounced auditory distraction effect when the stimulus sequence violates local and specific expectations that have been developed during prior exposure to the regular auditory sequences. Third, violations of global expectations based on information stored in long-term memory also capture attention, but only to a relatively small degree. Furthermore, the effect of attentional capture is transient; habituation occurs to initially unexpected steady state endings.

## General Discussion

Experiments 1, 2, and 3 illustrate that behavioral auditory distraction is more strongly determined by the violation of local expectations about the continuation of a to-be ignored auditory sequence than by the number of local changes in the irrelevant stream. The present results are consistent with previous studies showing that expectation violations are more important than the number and degree of local auditory changes in the stimulus material [17], [31], and they extend these earlier findings in several ways. First, the present study generalizes these findings to more complex and naturalistic stimuli (melodies and sentences). Second, previous studies focused on the effects of a single deviant event, whereas the present study extends these findings to whole sequences of repetitive steady state stimuli that violate expectations. Third, the data reported here show that the expectation violation effect does not only affect the encoding of to-be-remembered items, but also their maintenance in working memory (Experiment 3). Fourth, the opportunity to develop a neural model of the specific auditory distractor sequence is a necessary condition for an expectation violation effect large that is enough to counter the effect of local changes in the distractor material (Experiment 4). Although the violation of more global expectations based on participants' long-term knowledge causes a transient attentional capture effect, it is too small to offset the effect of the distractor material's reduced acoustic variability.

The results are important under an applied as well as under a theoretical perspective. From an applied standpoint, the present results can be considered relevant for determining the disruptive potential of auditory distractors. The present results suggest that a sound can contain many local changes, and still be only moderately distracting if it is highly predictable. In contrast, highly unpredictable sound sequences can be much more distracting, but distraction wears off quickly when the initially unpredictable sound sequence is repeated. This knowledge could be important, for instance for the design of alarm sounds for which the auditory stimulus' capability to capture attention is a desirable property. These sounds should not only contain a large number of local changes, but also should be highly unpredictable in order to boost their attention-grabbing potential. From a theoretical standpoint, the present results also contribute to the evaluation of working memory models that make conflicting predictions about the relative role of local changes and violations of expectations for the disruption of working memory processes.

For some decades, the object-oriented episodic record model was the standard model for explaining auditory distraction (see [47], [48]). According to this model [49], the disruptive potential of irrelevant sound on serial recall is solely determined by local changes in the distractor material. The model makes the very strong assumption that attentional processes are not involved in auditory distraction. Instead, the model implies that irrelevant sounds are preattentively processed, and this obligatory processing interferes with the short-term maintenance of the to-be remembered items because both types of processing overload shared processing resources. More specifically, it is assumed that the auditory stream is segmented into separate objects whenever local changes in the stimulus material are detected. The serial order of these auditory objects is automatically registered, which interferes with the maintenance of the order of the visual target items. An important prediction of this model is that the predictability of the auditory distractor sequence has no effect on auditory distraction [29], which is inconsistent with the present results.

Based on previous observations that auditory deviants disrupt serial recall (e.g., [32]) and that non-phonological distractor properties such as valence affect the degree of disruption (e.g., [45]), the O-OER model has been superseded by the duplex model of auditory distraction [30], [31], which maintains the assumption that the disruption of the rehearsal of the target items is caused by preattentional automatic interference of the processing of order information. In addition the model allows for the disruption of serial recall when highly distinct deviants capture attention. However, the model implies the assumption that attentional capture only interferes with the encoding of the target items, and should therefore not affect the maintenance of information in short-term memory. There are some aspects of the present results that are perfectly consistent with such an approach, while others are somewhat in conflict with it. Consistent with the duplex model's assumption that interference from expectation violation and interference from local changes rely on different mechanisms, the results of Experiment 4 show that both expectation violation and local changes interfere with serial recall. Habituation was observed to the disruptive effect of sequences comprising unexpected steady state endings, whereas the disruptive effect of the local changes in regular (variable) melodies and sentences remained constant. This is fully in line with the duplex model and its idea that there are separate forms of auditory distraction. It appears as if the disruptive effects of expectancy violations and those of local changes can be dissociated by habituation across trials. Care must be taken, however, that in each trial a different to-be-ignored sequence is played. Recently, it has been shown that the disruptive effects of complex distractor sequences such as the one used in the present study (melodies, speech) become smaller when the same sequence is presented repeatedly [40], while those of the same two alternating spoken words typically remain constant across trials (see also [29], [36]). In combination with the results reported here, this could be explained with a gradual reduction of the degree to which these stimuli violate expectations. Whereas a speech sequence, for instance, is relatively unpredictable (i.e., it does not provide a fixed set of rules from which regularities can be extracted at the first encounter), sequences of homogenous words, in contrast, which are designed to be as similar as possible with respect to length, intonation, loudness, and timing might simply be too predictable from the outset to allow for a benefit from repeated exposure. While the idea of two separate forms of auditory distraction seems to be applicable to the deviation effect and the changing state effect with simple distractor material (cf. [30], [31]), attributing disruption by complex sounds to one or the other is problematic. Likewise, the results of Experiment 3 are somewhat different from what the duplex model would predict. Unexpected steady state endings disrupted performance even when the distractor repetitions occurred after the presentation of the target items during a retention interval. This finding is inconsistent with the duplex model's assumption that attention capture selectively interferes with the encoding of the to-be remembered items, but has no effect on retention. In addition, this finding stands in contrast to a finding reported by Hughes et al. [31] in which a single delayed distractor in an otherwise regular sequence disrupted serial recall performance markedly when being played during the presentation of the item list, but had no effect in a retention interval. In the attempt to explain this discrepancy, one aspect seems to be particularly relevant: Hughes et al. [31] manipulated the presentation time of the distractors (encoding vs. retention) not within a single experiment, but across two experiments, only one of which comprised a retention interval. This way, participants in their Experiment 2 (in which no effect of attentional capture was obtained) had to maintain the to-be-remembered items twice as long as participants in their Experiment 1 (in which an attentional capture effect was found). As a consequence, the effect of auditory distraction was generally decreased in their Experiment 2 relative to their Experiment 1, and this general decrease may also have reduced the chances of finding an effect of a single attention-capturing event. One plausible reason as to why this may have been the case is that participants were able to take advantage of the long retention interval by rehearsing the previously presented target items 9 s instead of 3 s (as in Experiment 1) before the auditory deviant was played, which may have rendered the representation of the sequence more stable. There is reason to assume that with increasing length of retention, rehearsal becomes more automatized and therefore less vulnerable to attentional distraction (e.g., [50]). Hence, it seems that this previous study does not lead to a clear conclusion about the locus of the capture effect at encoding. To our knowledge the present experiment is the first in which the presentation of the auditory capture event has been manipulated within a single experiment without confounding presentation time (encoding vs. retention) with the length of the retention interval. The results suggest that attentional capture can indeed disrupt the maintenance of the items in memory (see [40] for a similar finding across experiments).

Further, the present results are consistent with models that assume that expectation violation can elicit an attention switch to the auditory modality, which may disrupt ongoing cognitive processing, such as Näätänen's model of attention and automaticity in audition [8], and the embedded-processes model [16]. Moreover, the present results shed light on the nature of the attention-capture mechanisms. Nöstl et al. [17] distinguished between the local changes account and the expectation violation account of attentional capture. According to former, auditory deviants capture attention when they differ from the immediately preceding stimulation, or occur with a low probability in the sequence. The present results showing that repetitive changing state sequences caused more interference than highly variable sequences in Experiments 1, 2, and 3 are clearly inconsistent with the local changes account, and strengthen the expectation violation account, according to which auditory distraction is primarily determined by the degree to which the to-be ignored sounds violate predictive rules that govern the auditory stream. Furthermore, the present series of experiments also allows us to determine the relative importance of local and specific in comparison to global and unspecific expectations for attentional capture effects. Experiment 4 demonstrates that attentional capture depends on local expectations. Consistent with previous studies [7], the expectation violation effect was markedly reduced when there was no opportunity to develop a neural model of the specific auditory distractor sequences (see also [33], [36], [51]). The violation of global expectations based on general knowledge about the continuation of melodies elicited only a transient attentional capture effect that was not large enough to offset the effect of the regular sentences' variability. Notwithstanding the above, less specific expectations had a limited influence on attention capture, too. In all experiments, participants habituated to the occurrence of steady state endings although a different melody or sentence was used in each trial. This shows that the cognitive system is flexible enough to adapt not only to specific auditory events, but also to abstract regularities in the stimulus material such as the occurrence of stimulus repetitions. Together, the findings suggest that auditory distraction is primarily determined by situation-specific abstract expectations, with more general expectations and local changes playing a minor role. Despite being more complex than a unitary account, such a conceptualization is consistent with a range of recent evidence on auditory distraction [5], [33], [52].
